# Supplementary material for: Are carbon emissions trading and green financial instruments synergistic? -Comprehensive quantitative research based on content analysis
Source: PLoS One. 2024 Mar 7;19(3):e0298601. doi: 10.1371/journal.pone.0298601 (PMC10919601; doi:10.1371/journal.pone.0298601)
Supplement: S1 Data — (ZIP) [file pone.0298601.s001.zip › Data/地级市Z_F工作报告.zip!/╣π╢1⁄2/╜╥╤⌠╩╨/└┤╫╘╣1⁄2╓┌║┼í╛┬φ┐╦╩2╛▌═°í┐_2014.docx]

各位代表：

现在，我代表市人民政府向大会作工作报告，请予审议，并请市政协各位委员和其他列席人员提出意见。

2013年工作回顾

过去一年，我们坚决贯彻省委、省政府的决策部署，在市委的正确领导下，在市人大及其常委会和市政协的监督支持下，紧紧围绕“打造粤东发展极，建设幸福新揭阳”的核心任务，牢牢把握“强化效率、彰显公平、惠民执政、绿色发展”的施政理念，改革创新，奋发进取，顽强拼搏，沉着应对错综复杂经济形势的挑战，经受住百年不遇自然灾害的考验，在建设粤东经济强市和粤东上善之区的征程中迈出了坚实的步伐。

（一）以经济协同增长推动提速增效。着力增投资、扩消费、稳出口，推动“三驾马车”协调发展。全年生产总值1605.35亿元，比上年（下同）增长14.5%，跃居粤东第一。加快中委广东石化炼油、中海油粤东LNG一体化、中电投通用码头等重点项目建设，创建中德金属生态城，固定资产投资829.39亿元，增长27.5%。组织企业赴俄罗斯、波兰等国展销产品，举办揭阳产品全国行（昆明）展销活动、第三届玉文化节、普宁国际内衣博览会、揭货“网上行”活动，开拓国际国内及“网络”市场，社会消费品零售总额657.66亿元，增长15.2%；外贸出口43.8亿美元，增长15 %。工业持续快速增长，规模以上工业增加值860.79亿元，增长23.8%；工业用电量增长14%。质量效益明显提升，地方公共财政预算收入66.69亿元，增长17.6%；各项存款、贷款余额分别增长16.67%、17.82%。

（二）以产业组织化建设增强发展动力。深入实施“6166”计划，加强产业组织化建设，出台扶持产业发展政策，确立六大千亿产业建设目标。金属、服装、医药、石化产业增加值分别增长27.2%、26.4%、14.3%、17.6%。培育电商产业，创建国家电子商务示范城市，军埔成为闻名全国的“淘宝村”；电商销售总额增长38%；邮政业务总量增长52.7%。建成全国首个中药材大宗交易平台。仙宜岱成功登陆新三板。商务旅游示范区建设取得新成效，旅游总收入突破百亿元大关。

（三）以平台建设助推转型升级。加快空港、海港经济区和20个产业园区规划建设，打造产业聚集平台，中德金属生态城成为省重大发展平台。与中科院、省科技厅共建科技创新试验区，南方（揭阳）科技成果交易平台上线运行，新增高新技术企业5家、企业研究院1个、院士工作站2个、博士后工作站2个、省级工程研发中心10个，技改投资超百亿元。建设粤东金融强市，与人行广州分行共建金融试验区，21个重点项目获银行授信510.1亿元。培训城镇在岗职工3万人次，建设人才公寓，搭建粤东人力资源高地。

（四）以扩容提质加速城镇化进程。调整行政区划，启动市城规和土规修改评估工作。揭阳新区获省批准，空港新城、揭东新城、玉都新城起步建设。创建省文明城市，建成数字城管系统和进贤门大道东段、莲花大道南段等市政项目，道路畅通工程上升为二等。获批“三旧”改造项目7宗。揭阳潮汕机场获批一类口岸，实现对台直航；厦深高铁建成通车；潮惠、汕湛、揭惠高速公路揭阳段动工建设，改造国省道80公里。完成东山截污工程等省污染减排计划项目33宗。建设金属表面处理中心，谋求电镀产业“零排放”。开展新一轮绿化揭阳大行动，建成生态景观林带176公里、森林碳汇工程27.43万亩、绿色单元438个，市区新增公共绿地面积12.5万平方米。

（五）以国际合作拓展发展格局。抢抓全球资源重新配置的机遇，实施以德国为重点的国际合作。金属联合会与德国家族企业基金会等9个协会深度合作；与阿德勒斯霍夫科技园达成园区管理合作意向；弗劳恩霍夫、巴塞罗那工程设计学院等5家科研机构入揭发展；与达姆施塔特工业大学共建绿色排放系统；与达姆施塔特应用科技大学共建产业大学；引进德国退休工程师担任企业顾问；法兰克智能管网公司等11家企业正式落户。服装产业与法国合作，外贸企业协会与伊朗合作，传统产业走进非洲，与联合国工业发展组织共建绿色工业示范区，揭阳正成为全球资源配置链的新节点。

（六）以惠农增收引领“三农”发展。加大“三农”投入，促进农业增效、农民增收、农村发展。20个“一镇一品”示范镇农业总产值增长8.9%，主导产品产值增长16.5%，普宁高埔镇被评为全国“一镇一品”示范镇。动员全市力量抗击超强台风和特大洪涝灾害，复产重建工作取得重大胜利。投入水利资金8.06亿元，榕江大围达标加固工程、惠来中东部供水工程等一批水利重点项目进展顺利。实施“乡贤乡村建设”工程，筹集34.54亿元投入农村基础设施建设。落实扶贫开发资金2.61亿元，实施帮扶项目1444宗，完成农村危房改造4735户。

（七）以社会事业发展增进民生福祉。扎实走民生引领型发展路子，投入民生事业资金125.12亿元，竭力为民办实事、干好事、谋福祉。认真办理省十大民生实事和市30件民生实事。9个镇（街道）获省授予教育强镇称号，高考一批上线人数增幅达30.1%。筹集大学生发展基金887万元，资助贫困学生2190人。市人民医院成为中山大学非直属附属医院，慈云医院实现引资托管，普宁康美医院建成投入使用，城乡居民基本医疗保险参保率达96.5%。新增全国重点文物保护单位3处，举办“乡乡乐起来”523场。市广播电视中心、档案馆新馆等文化设施投入使用。出版发行首部《揭阳市志》。新增城镇就业3.58万人，城乡居民养老保险参保208.12万人。建设保障性住房3028套。推进平安揭阳建设，建成视频监控点3万个、治安卡口100个，成功处置上浦村群体性事件，综治信访维稳和消防工作得到加强，安全生产形势持续好转。

（八）以为民务实清廉强化政府作为。推进行政体制机制改革，竭力反腐倡廉，建设为民务实清廉政府。市、县（市、区）政务服务中心和网上办事大厅建成投入使用，初步建成四级政务服务体系。开展企业登记注册改革试点，改革食品药品监管机构、卫生和计生机构。强化行政监察和审计监督，发挥民营经济市长特派员作用，开展“庸懒散奢浮”整治，查处违法违纪507人、行政问责336人。人大代表、政协委员建议提案办理满意率均达到100%。

此外，统计、气象、地震、供销、物价、体育、普法、海洋渔业、妇女儿童、人口计生、残疾人、双拥、人民防空、人民武装、国防动员、民族宗教、港澳台、社科、文联等工作也取得了新进步。

各位代表，成绩来之不易，发展态势鼓舞人心，这是贯彻落实省委、省政府决策部署的结果，是市委正确领导、科学决策的结果，是市人大、市政协和社会各界有力监督、大力支持的结果，是全市上下团结奋进的结果。在此，我代表市人民政府，向全市干部群众致以崇高的敬意！向关心支持揭阳改革发展的各界朋友、港澳台同胞、海外侨胞和国际友人表示诚挚的感谢！

同时我们也清醒地认识到，我市还存在不少问题和挑战：一些领域市场壁垒依然存在，深化改革任重道远；环境资源约束依然趋紧，环保治污、用地治违力度仍需加大；财政运转压力较大，民生投入仍不足；观念滞后、人才缺乏依然是制约发展的“瓶颈”；权力运行仍需规范，廉政建设必须加强。对此，我们将高度重视，采取扎实措施，认真加以解决。

2014年工作安排

2014年是全面深化改革的第一年，也是完成“十二五”规划目标任务的关键一年。今年政府工作总的要求是：深入贯彻落实党的十八大、十八届三中全会、中央经济工作会议、省委十一届三次全会和市委五届六次全会精神，紧紧围绕“打造粤东发展极，建设幸福新揭阳”的核心任务，以加快发展为首务，以维护稳定为底线，以改革创新为引领，着力发挥市场决定性作用，着力转变发展方式，着力产业培育，着力创新社会管理，着力全面深化改革，努力建设粤东经济强市和粤东上善之区，奋力开创揭阳跨越崛起新局面，争当粤东西北地区加快发展排头兵。

今年主要预期目标是：生产总值增长15%以上；规模以上工业增加值增长24%；社会消费品零售总额增长18%；固定资产投资增长30%以上；外贸出口增长16%，实际利用外资增长15%；地方公共财政预算收入增长17.5%以上；居民消费价格涨幅控制在3%左右，城镇登记失业率控制在3.5%以内；城镇居民人均可支配收入增长13%，农村居民人均纯收入增长15%。重点抓好以下工作：

（一）攻坚克难搞建设

举全市之力建设交通基础设施、产业园区和中心城区，加快空港、海港投资开发，夯实经济发展基础。

狠抓空港海港建设。优化空港海港规划，明确两港建设的着力点，集聚资源，加快建设。支持空港经济区创建粤东区域电子商务快递物流中心、潮汕传统产业批发会展中心；提升玩具、模具、石材等特色产业，培育生产性服务业。指导海港经济区产业建设，创新大南海石化工业区体制机制，盘活土地资源，集聚金融要素，加快基础设施建设；出台招商引资政策，实现有目标、有计划的精准招商，力争一批石化骨干企业落地开工，形成新一轮产业集聚。

狠抓重点平台建设。加快中德金属生态城建设，在办园模式上形成可持续、可复制的制度安排，在全产业链建设上探索要素资源聚合增效的路子，在绿色工业、产城融合上创建新模式。推动高新区与蓝城区形成一体化管理新格局，实现互促共进发展。发挥普宁服装联合会作用，规划建设中法纺织服装生态城。进一步明确揭东、英歌山等工业园区产业主题，集聚优化资源配置，形成动力强劲的发展平台。

狠抓城市扩容提质。强化建设揭阳新区，优化提升老城区。新区要着力于生产、生活、生态“三生”融合，坚持产业立区，培育支撑性产业，科学配置教育、医疗、文化、体育等公共资源；坚持生态立区，创建大树底下的幸福生活。老城区要梳理提升特色经营街区，培育社区经营龙头，培训街区创业群体，形成街区创业创富体系；优化社区公共服务配套；建设绿色街区和文化、体育街区。着力于空港和高铁交汇区、高铁站口拓展区、高速出口辐射区的开发建设，使之成为扩容提质的重要载体。

狠抓交通设施建设。完善揭阳潮汕机场配套设施，加快揭阳空铁换乘中心和机场、高铁周边路网建设，实现交通立体综合对接。规划申报惠来港区公共航道，加快中石油、中海油、中电投等码头建设，推进与荷兰鹿特丹港务局合作。整合提升榕江港口群，加强榕江航道整治，培育榕江航运物流带。抓好广梅汕铁路改线工程、揭阳港疏港铁路、粤东城际轨道揭阳段规划建设。加快汕湛、潮惠、揭惠等高速公路揭阳段建设，改造国省道70公里、地方公路500公里。

（二）持之以恒育产业

产业是地方发展的动力源，是民众就业的主载体。坚持产业强市，继续实施“6166”计划和八大暖企行动，扎实推进产业“登高”工程和引进工程，打造揭阳产业升级版。

强化产业体系建设。围绕六大千亿产业建设目标，形成坚实的产业框架。一是深化产业协会建设，实现产业资源互补共享，形成联系紧密、组织化程度高的产业合作体系；扶持培育一批龙头骨干企业，形成产业梯队。二是实施产业“登高”工程，继续举办榕江企业家培训班和经营、管理、生产骨干训练营，为龙头企业配备外脑和洋顾问，形成产业转型升级的核心力量；鼓励引进创建研发设计、物流仓储、品牌培育营运、人力资源等生产性服务机构，完善产业链条；建设产业金融，实现业业有投资基金、有小贷公司、有担保公司；培育金融租赁公司；加快产业征信体系建设；组织产业“走出去”，给予对外合作示范企业融资贴息；全要素孵化一批上市企业。三是创新产业建设经营方式，推广土地、厂房、设备租赁的轻资产运营模式，创建产业孵化器，推动科技成果快速产业化。四是实施产业引进工程，确定产业引进目标，建立引进平台，出台引进政策，配置产业资源，创立产业落地服务体系，开展产业引进大竞赛，快捷、精准、有效地形成产业增量。

深度开展国际合作。以中德合作促进观念转变、资源集聚、转型升级，以高端合作的先人一步，实现揭阳技高一筹。一是出台金属生态城规章制度，试行“负面清单”管理模式。引入德国园区管理模式。加快金属生态城建设，确保年内主干道路、五大中心、专家服务基地建成投入使用，打造新型国际合作平台。二是加快金属生态城原材料及制品营销、研发设计、生产制造、城市矿山、人力资源及产业金融六大平台建设，举办中欧科技与设计交易会，创建国家级国际科技合作区，实现高端要素充分集聚，实现资源的溢出与辐射。三是加强与德国及欧盟国家行业协会合作，使金属生态城成为相关产业进入中国的门户与平台。四是重点服务法兰克智能管网、艾萨医疗器械、中德科技孵化器等入园项目建设，使之迅速投产达产。五是将金属生态城模式复制至中法纺织服装生态城、海峡食品药品合作区。六是扩大国际合作领域，推动服装产业与法国、制鞋业与意大利、港口建设与荷兰、玉产业与缅甸合作，办好非洲国家青年电子商务领袖培训班，与非洲深度合作，同时开展走进伊朗活动，形成揭阳产业国际合作新格局。

大力发展县域产业。指导县（市、区）梳理产业发展重点，优化配置要素资源，形成特色产业集群。普宁市重点发展服装、医药产业，推动产业合作、产业升级，组织商贸企业“上网络”，创建中法纺织服装生态城。榕城区重点把握特色经营街区的整理提升，打造街区创富体系，在金融业、文化创意产业、城市旅游业及鞋业研发设计、会展上求突破。揭东区结合中德金属生态城建设，发展先进制造业；结合军埔村、中德风情小镇等建设，发展壮大电子商务等现代服务业。蓝城区推动玉文化产业转型升级；结合医药学院的迁建发展医疗健康产业；与高新区共建海峡食品药品合作区；创建金融创新试验区。空港经济区在粤东地区快递产业、产品集散批发会展上抢先布局，建设玩具创意、模具、石材产业集聚区。揭西县着力发展生态旅游业、绿色农业、农村金融业，创建电线电缆转型升级园区。惠来县突出发展临港工业、滨海旅游业、海洋产业。大南海石化工业区大力发展石油炼化、精细化工产业。普侨区对接普宁，大南山侨区对接大南海石化工业区和惠来县，发展生产性服务业、绿色生态健康产业。县域产业的建设要把握区域优势、顺应发展态势、遵循市场规律，坚定发展主题，跨代周期性培育，最终形成产业集群、产业高地。

形成电商先发优势。电子商务是零距离对接消费群体的产业立交，是升级版的实体经济新形态。围绕促进商贸业及传统制造业转型升级的双目标，结合创建国家电子商务示范城市，大力实施“8610”计划：以建设电商样板军埔村、组建顾问团队、创建电商研究所、筹建电商学院、出台全市电商发展规划、制订工作实施方案、出台扶持政策、筹建产业基金“八个一”为切入；以建设军埔为中心的揭东集聚区、跨境电商为方向的中德集聚区、粤东快递物流为基础的空港集聚区、服装医药产业为重点的普宁集聚区、街区特色经营为依托的榕城集聚区、玉文化产业为载体的蓝城集聚区等六个重点区为着力；以十万电商骨干大培训为重要手段，对电商产业进行率先布局，形成揭阳电子商务先发优势，建设潮汕产业的“网络港”。

加快重点项目建设。对已落户的重点项目，按照“一个领导、一套人马、一揽子措施、一个时间周期”开展全方位服务。对中委炼油等重点项目，集中全市之力，有针对性地解决困难、提供配套，以服务促建设、促效益。制定在揭重点项目服务方案，从土地、金融、人力资源、社会治安等方面全力支持，营造“风和日丽”的产业成长环境。充分发挥民营经济市长特派员作用，集中民意民智服务项目建设、促进产业育成。

（三）深入扎实强“三农”

抓好农村综合改革和“一镇一品”建设，稳定农业生产，增加农民收入，改善农村环境，夯实“三农”工作基础。

提升“一镇一品”工程。围绕“创品牌、拓销售、促种植、出效益”，组建“一镇一品”联盟，打造十大示范乡镇、十大示范企业和十大示范合作社，增强“一镇一品”的示范带动力。支持普宁高埔等“一镇一品”产业园区规划建设。加强骨干力量培训，致力培养农业领军人物。加强与省农科院等院校合作，共建“一镇一品”研究院。创建“一镇一品”产业基金，发展农业保险，以金融保险助推农业发展。依托机场、高铁站及军埔村，建立“一镇一品”线上线下营销平台，让农产品卖好价，让农民增效益。

加强农业基础建设。推进榕江大围达标加固、惠来中东部供水、普宁白坑湖水库综合整治等重点水利工程，抓好江海堤防、山塘水库和水闸等修复加固工程，提高防灾减灾能力。谋划引韩供水工程。抓好高标准农田建设，完善农田排灌系统，推动丢荒耕地复耕复绿，提升高产稳产农田比重。继续实施“乡贤乡村建设”工程。扎实推进扶贫开发“双到”工作。

完善农村基本制度。坚持稳定土地承包关系，依法保障农民对承包地使用、收益、流转及承包经营权抵押、担保权利。探索农村土地集体所有制的有效实现形式，落实集体所有权、稳定农户承包权、放活土地经营权。完成村级换届工作，推进基层民主自治。

（四）抓铁留痕促生态

把环保生态作为增进民生福祉、促进永续发展、增强区域竞争力的重要举措，严守生态红线，偿还生态负债，保青山保绿水保洁净空气。

开展环保为民大行动。铁腕重典治污，营运金属表面处理中心，打造中国首个“零排放”电镀产业集群，变黑色电镀为绿色电镀。启动建设酸洗、漂染生态园。筹资6.3亿元建设一批环保设施，确保市和揭东污水处理厂二期以及炮台、里湖等9个污水处理厂建成投运，开工建设市区和普宁垃圾焚烧发电厂，完善各地垃圾填埋设施。按照“死保龙江、整治练江、提升榕江”原则，开展重点流域水污染综合整治，坚决关闭取缔环保违法企业。重拳整治PM2.5、医疗废物、电子废物和农业污染源，切实改善环境质量。推进与联合国工业发展组织共建绿色工业示范区。

开展绿化揭阳大行动。牢固树立“人树一体”理念，重点抓好森林进市区、大树入门庭、生态作屏障、江海建林带、新区建公园等城乡绿化“五大建设”，努力做到见缝插绿、门前植绿、沿路布绿、满山播绿、全城尽绿。建设中德森林公园、潮汕林科基地，新增造林13万亩，森林覆盖率提高至54.98%。

开展保卫资源大行动。坚持“以亩产论英雄”，落实保障产业用地政策，严厉打击圈地囤地行为，遏制违法违规用地。加快“三旧”改造，提高集约节约用地水平。清理沿海沿江港口岸线，促进有限岸线资源合理高效利用。加强海洋资源保护和开发，树立“蓝色国土”保护理念，建设“美丽海湾”。

（五）真心实意惠民生

坚持民生引领，大力发展社会事业，让发展成果惠及广大人民。

深化教育创强工作。实现榕城和揭东等6区创建教育强区目标，新增教育强镇36个，义务教育标准化学校覆盖率达到66.9%。为揭阳一中增编增资，建设潮汕地区最具培养力的名校。与北京外国语大学、北师大等高校合作，引入国际双语学校，促进优质教育资源短平快集聚。深化与华师大战略合作，继续实施师资队伍培训提升工程。加快与德国达姆施塔特应用科技大学合作建设中国首家双元制职业大学，推动揭阳职业技术学院实现“区域化、市场化、开放化、品牌化”办学目标，组建揭阳职教协作组织，建设南方高技能人才集聚区。

做好卫生计生工作。密切与中山大学、南方医科大学等战略合作，引入优质医疗资源。建设市人民医院门急诊住院综合楼和各县（市、区）人民医院住院楼，筹建揭阳医药学院，形成一批具有区域影响力的专科医院。培育引进基层全科医生，深化骨干医生“下基层、带徒弟、强医德”活动，健全基层医疗卫生服务体系。组建医疗健康产业联盟，建设医疗健康产业高地。抓好蓝城区基层医改保险试点，推进医疗卫生信息化建设。加强人口计生管理，稳定低生育水平。

繁荣发展文化事业。弘扬新时期揭阳精神，唱响“中国梦”的主旋律。充分挖掘文化资源，培育文化人才，推进揭阳学宫、进贤门城楼等修缮工程，建设国际玩具创意基地、南方（揭阳）潮汕文化产业园和阳美玉雕文化（创意）产业基地。筹建市文史研究馆，完成市、县两级文化馆、图书馆、博物馆建设任务。继续开展“乡乡乐起来”等群众性文化活动。

推进其他社会事业。加大劳动监察执法力度，强化社保扩面征缴。办好第六届市运会，推动全民健身。大力发展健康养老服务业。做好新闻出版、广播电视、供销、普法、气象、地震、国防动员、人民防空、双拥、民族宗教、外事侨务、社科、文联、档案等工作。

（六）尽心竭力办实事

办好十件民生实事。一是提高百岁老人津贴标准，每人每月从200元提高至400元，各县（市、区）适当提高80岁以上老人津贴标准，建设长寿之乡；二是创建农村留守老人、儿童关爱基金，建设留守老人关爱中心、留守儿童学校和农村应急避险中心；三是筹资1亿元开展城市大绿化行动，建设社区绿岛、街道绿廊、沿江绿带、城市森林、湿地公园，启动黄岐山整治植绿工程，建设绿色岐山，实现森林进城围城；四是筹集1亿元，改造榕华大桥等一批危桥；五是结合“乡贤乡村建设”工程、农村清洁工程，建成一批村级垃圾收集点、镇级转运点、区域统一处理中心；六是引进培养乡镇卫生院全科医生，给予全市乡镇卫生院全日制本科学历以上医生每人每月500元人才津贴，让人民群众有贴身的健康保护神；七是组建市轻舟支队、县（市、区）轻舟分队、重点乡镇轻舟小队，完善基层救灾应急队伍；八是启动建设粤东（揭阳）残疾人集中就业培训基地；九是规划建设面向社会的揭阳一中文化艺术中心、科技中心；十是建设固体废弃物处理与资源回收再利用中心。

（七）千方百计保稳定

防微虑远，着力解决经济社会突出问题，确保和谐稳定。

保障经济安全运行。抓好经济监测预警和统计分析，牢牢掌握经济工作主动权。推进涉税信息共享，提高依法治税水平。强化财政预算刚性约束，严格控制“三公经费”，集中财力谋发展、惠民生。加强社会信用体系和市场监管体系建设，严厉打击走私贩私、制假售假等行为，维护市场经营秩序。加强粮油储备调节，抓好“菜篮子”工程建设，稳定民生物价。

解决重点领域问题。加强政府债务审计监督，严格政府举债程序，防范和化解地方政府性债务风险。加强经济重点领域管理，严厉打击非法金融活动，维护辖区金融安全稳定。加强寄递物品安全管理。加大食品药品监管力度，确保人民群众“舌尖上的安全”。

维护社会和谐稳定。加强警察队伍建设，让人民警察站得正、靠得住，刚正不阿为人民。深化平安揭阳建设，完善社会治安视频监控系统，深入开展治赌、治毒、治假、治乱“四治”行动，着力解决治安突出问题。加大“打非治违”力度，加强消防设施建设，预防和遏制各类生产安全事故发生。高度重视群众来信来访，主动排查化解社会矛盾。

（八）敢为人先谋改革

把改革作为头等大事，积极稳妥地推进各项改革，增创发展新优势。

深化经济体制改革。破除房地产、建设工程、交通运输等领域隐性市场壁垒，按照“非禁即入”原则，运用“负面清单”管理模式，放宽民间投资领域和准入限制，激发社会资本活力。创新城市建设投融资体制，推动城市资源市场化运作，实现城市自我积累、滚动发展。以中德金属生态城为试点，创建知识产权保护示范区。

深化社会体制改革。创新教育、卫生、文化等体制机制，健全多层次公共服务体系。改进社会治理方式，培育发展社会组织，加大政府购买社会服务力度。改革城市管理体制，最大限度向各区下放市级管理权限。健全基层综合服务平台，整合社会服务管理资源，推行“一站式”便民服务。

深化行政体制改革。推进新一轮市县政府机构改革，转变政府职能，抓好中德金属生态城服务平台和行业协会依法承接政府经济管理职能的试点。大力推进行政审批制度改革，完善四级服务平台，建立办事工作日和告知承诺机制，提高机关行政效能。

加强政府自身建设，自觉接受人大、政协和社会各界监督，广泛听取民主党派、工商联、无党派人士意见，推进依法行政、科学决策。全面落实政府系统党风廉政建设责任制，严格执行中央“八项规定”，加强行政监察和审计监督，加大预防和惩治腐败力度。贯彻“照镜子、正衣冠、洗洗澡、治治病”的总要求，深入扎实开展党的群众路线教育实践活动，以严的标准、严的措施、严的纪律坚决反对“四风”，建设法治政府、服务型政府。

各位代表！让我们紧密团结在以习近平同志为总书记的党中央周围，在省委、省政府和市委的坚强领导下，志存高远，脚踏实地，真抓实干，开拓创新，一步一个脚印，为实现中华民族伟大复兴的“中国梦”和我省“三个定位、两个率先”总目标作出新贡献！
